# 2329: Psychosocial risk factors mental health symptoms, and intervention preferences of Latino immigrant parents presenting to a pediatric clinic

**DOI:** 10.1017/cts.2017.260

**Published:** 2018-05-10

**Authors:** Rheanna Platt, Elisabet Arribas-Ibar

## Abstract

OBJECTIVES/SPECIFIC AIMS: (1) To assess the prevalence of mental health symptomatology (depressive symptoms, anxiety symptoms, PTSD symptoms, and problematic alcohol use) and psychosocial risk factors for mental health disorders (low social support, immigration stress, acculturation, and marital/partner discord), and their association with immigration status, health care access and contextual risk factors in Spanish-speaking parents of young children (ages 0–5) who attended a well-child visit. (2) To explore acceptability of screening for and discussing parental distress in the pediatric primary care setting, and parental acceptability of a group well-visit format to address both psychosocial risk factors and mental health symptoms in this population. METHODS/STUDY POPULATION: Latino immigrant parents (n=100) of children ages 0–5 attending well-child visits at Johns Hopkins Bayview Children’s Medical Practice were surveyed between October 2015 and February 2016. The verbally administered survey included the Woman Abuse Screening Tool (WAST), AUDIT-C, Primary Care Post-Traumatic Stress Disorder (PC-PTSD) Screener, California Health Interview Survey (CHIS), National Latino and Asian American Study (NLAAS), Appraisal Support Subscale from Interpersonal Support Evaluation List (ISEL), Personal Health Questionnaire Depression Scale (PHQ-8), and Generalized Anxiety Disorder Scale (GAD-2). These questionnaires have been used in large regional or national surveys and most have been validated with US Latino populations. Positive screens were defined as PHQ-8>5 (mild depression or greater), GAD-2>3, AUDIT-C>3 for women and >4 for men, and PC-PTSD>3. Descriptive information and comparisons were obtained by χ^2^ and Student *t*-test. Study protocol will allow review of childrens’ pediatric records (n=100). From this sample, parents were separately recruited to participate in in-depth interviews (n=11 of 20 planned have been completed) further exploring both sources of parental distress, acceptability of screening for parental mental health symptoms in the primary care pediatric setting, and acceptability of a potential group-based well-visit model in the pediatric setting. RESULTS/ANTICIPATED RESULTS: Survey participants were 93.0% women, and predominantly<35 years of age. The vast majority (94.0%) were undocumented, recently arrived (<15 years ago) and reported poor or very poor English proficiency (75.0%). Most (84.7%) reported living with a partner or spouse (84.7%), and 58% reported partner relationship strain. In all, 71% reported poor social support. The prevalence of “screen positive” mental health symptoms was highest for depression (55%) and PTSD (35%), followed by anxiety (29%) and alcohol risk use (25%). Having depression was significantly higher (68.4%) (*p*<0.02) in participants with less education (<6 grade). Partner relationship strain was associated with a higher prevalence of depressive symptoms (59.3%) (*p*<0.03). Immigration stress (feeling guilty for leaving family and friends) was also significantly associated with depressive symptoms (58.1%) and PTSD (43.5%) (*p*<0.03). More than half of the participants (60.0%) with depression were not covered by any health insurance and 56.3% of those with depression reported not having been seen by a health care provider in the past 12 months. A high prevalence of symptoms was found in those with poor appraised social support: alcohol risk use (76.0%), depression (69.1%), anxiety (69.0%), and PTSD (68.6%). Among participants, those aged<30 years old and those with more children reported poorer appraised social support. Data from child medical records (including BMI, presence of feeding problems, referrals for social work, or mental health services) has been extracted but not yet linked to parent survey or interview results. Preliminary review of In Depth Interviews suggests that the most common reported source of stress among participants was related to finances, followed by documentation/legal status difficulties, access to childcare, and limited English proficiency. Some mothers also mentioned interpersonal violence and lack of access to healthcare as stressors. All mothers expressed an interest in a pediatric primary care based parent focused the majority of which indicated that a group based intervention would be acceptable, some mothers indicated they preferred a one-to-one intervention if mental health were to be discussed. Mothers seem preferential to social worker-led interventions compared with pediatrician-led, but most mothers were indifferent. Finally, mothers expressed low support from the Latino community in Baltimore. DISCUSSION/SIGNIFICANCE OF IMPACT: Results from this study suggest that this population of parents is experiencing a relatively high rate of mental health symptoms, low perceived social support, and limited access to their own source of care. This suggests that an intervention delivered within a primary care pediatric setting would have the potential to reach parents who might not otherwise interact with their own providers, and that there are an array of problems that could be targeted. Intervening with parents of young children has the potential to affect multiple child outcomes. A group intervention may target poor social support, though this format is not universally preferred. Next steps for this project include assessing the acceptability of and preference for various content components (ie, depression, parenting stress, legal issues) and linking parent data with child data (including developmental screening results, weight, feeding problems, and behavior problems).

